# STOP algorithm for bedside mechanical ventilation: Standardized, evidence-based management of critically ill patients

**DOI:** 10.17305/bb.2026.13288

**Published:** 2026-01-12

**Authors:** Oguz Kilickaya, Dimitrios Kantas, Nirmala Manjappachar, Baiyong Wang, Marko Nemet, Rana Gur, Yue Dong, Srdjan Gajic, Mirela Alic, Philippe R Bauer, Sumera Ahmad, Alice Gallo de Moraes, Alexander Niven, Richard A Oeckler, Amos Lal, Ognjen Gajic

**Affiliations:** 1 Division of Pulmonary and Critical Care Medicine, Mayo Clinic, Rochester, Minnesota, USA; 2 Department of Anesthesiology, Mayo Clinic, Rochester, Minnesota, USA; 3 Department of Pulmonary and Critical Care, Virginia Mason Medical Center, Seattle, Washington, USA; 4 Division of Pulmonary and Critical Care, Mayo Clinic, Jacksonville, Florida, USA

**Keywords:** Acute respiratory distress syndrome, autoPEEP, decision aid, mechanical ventilation, ventilator-induced lung injury, weaning

## Abstract

The COVID-19 pandemic revealed significant variability in mechanical ventilation training and bedside practices, highlighting the necessity for standardized, actionable protocols. This study aimed to develop the Standard Training and Operating Procedure (STOP), an evidence-based algorithm designed for managing mechanically ventilated critically ill patients and troubleshooting patient-ventilator interactions. Utilizing the Successive Approximation Model (SAM), we reviewed current guidelines and expert recommendations, created a minimum-viable prototype during a multidisciplinary “savvy start,” and refined it through seven iterative review cycles involving 33 frontline clinicians. The finalized tool underwent external evaluation via a Modified-Delphi process within the Checklist for early recognition and treatment of acute illness and injury (CERTAIN) network, engaging 50 clinicians from 19 countries across four continents, with a consensus threshold of ≥70%. STOP consists of eight sequential bedside checkpoints: abnormal vital signs/ventilator alarms, assessment of ventilation adequacy, elevated peak pressure, elevated plateau pressure, lung protection against ventilator-induced lung injury, risk of oxygen toxicity, patient-ventilator asynchrony, and readiness for spontaneous awakening and breathing trials. The Delphi agreement across these steps ranged from 82% to 96%, supporting the tool’s face validity and clinical relevance. STOP offers a practical framework to minimize practice variability and enhance the safety of mechanical ventilation; however, prospective implementation studies are necessary to assess its impact on adherence and patient outcomes.

## Introduction

Inconsistent quality in critical care practices has long been recognized as a primary barrier to improving patient-centered outcomes [1]. This issue is particularly pronounced in the management of mechanically ventilated patients, where variability underscores the need for standardized, evidence-based protocols to optimize care delivery [2]. Effective ventilator management is crucial for supporting respiratory function in critically ill patients; however, clinicians face challenges due to the wide variety of ventilators, multiple ventilation modes, and complex displays [3]. Additionally, the unequal distribution of respiratory therapists for ventilator management exacerbates these challenges [4]. This complexity often results in data overload and significant variability in practice, contributing to medical errors and inconsistent quality of care [5]. The COVID-19 pandemic further illuminated these challenges as intensive care units (ICUs) globally faced overwhelming demand, revealing disparities in mechanical ventilation training and practices across different regions [6].

Although previous research has emphasized the advantages of an evidence-based approach to mechanical ventilation, notable gaps persist in translating these findings into standardized protocols that balance modern complexity with the simplicity necessary for effective bedside application [7, 8]. Protocols that streamline decision-making by focusing on essential components with the highest impact on patient outcomes are most likely to succeed [9].

To address these gaps, we aimed to develop a “Standard Training and Operating Procedure (STOP)” that provides a structured approach for guiding the bedside management of mechanical ventilation in both common and critical conditions encountered by critically ill patients, while also assisting in troubleshooting patient-ventilator interactions using current guidelines and the latest expert recommendations for best practices.

## Materials and methods

**Figure 1.**
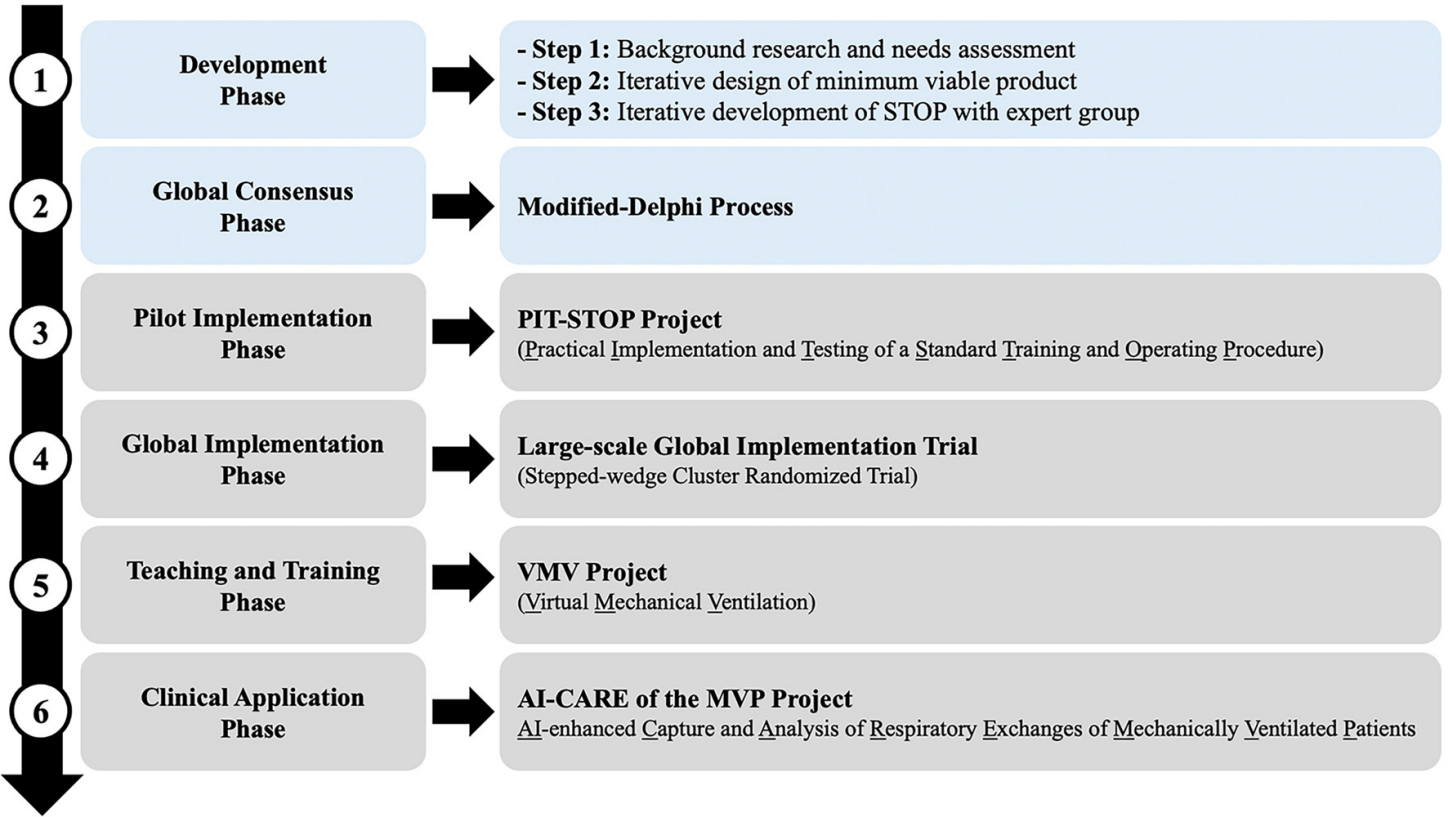
**Overview of the STOP project’s multi-phase approach.** This figure presents a comprehensive summary of the project structure, illustrating a stepwise sequence of phases that outline the progression of the STOP initiative. It details the transition from initial development activities to extensive validation and subsequent implementation in practice. The figure serves as a high-level roadmap of the major stages of the project, highlighting the planned progression from foundational work to pilot testing, larger-scale implementation efforts, concurrent educational components, and downstream clinical application pathways.

To achieve these objectives, the STOP Project employs a multi-phase approach, with each phase incorporating specific ancillary projects aimed at building, refining, testing, and deploying the STOP algorithm in both clinical and educational settings (Figure 1). This manuscript focuses on the conceptual development, instructional design framework, and iterative prototyping of the STOP algorithm. The study protocol received evaluation and approval from the institutional review board (IRB).

**Figure 2.**
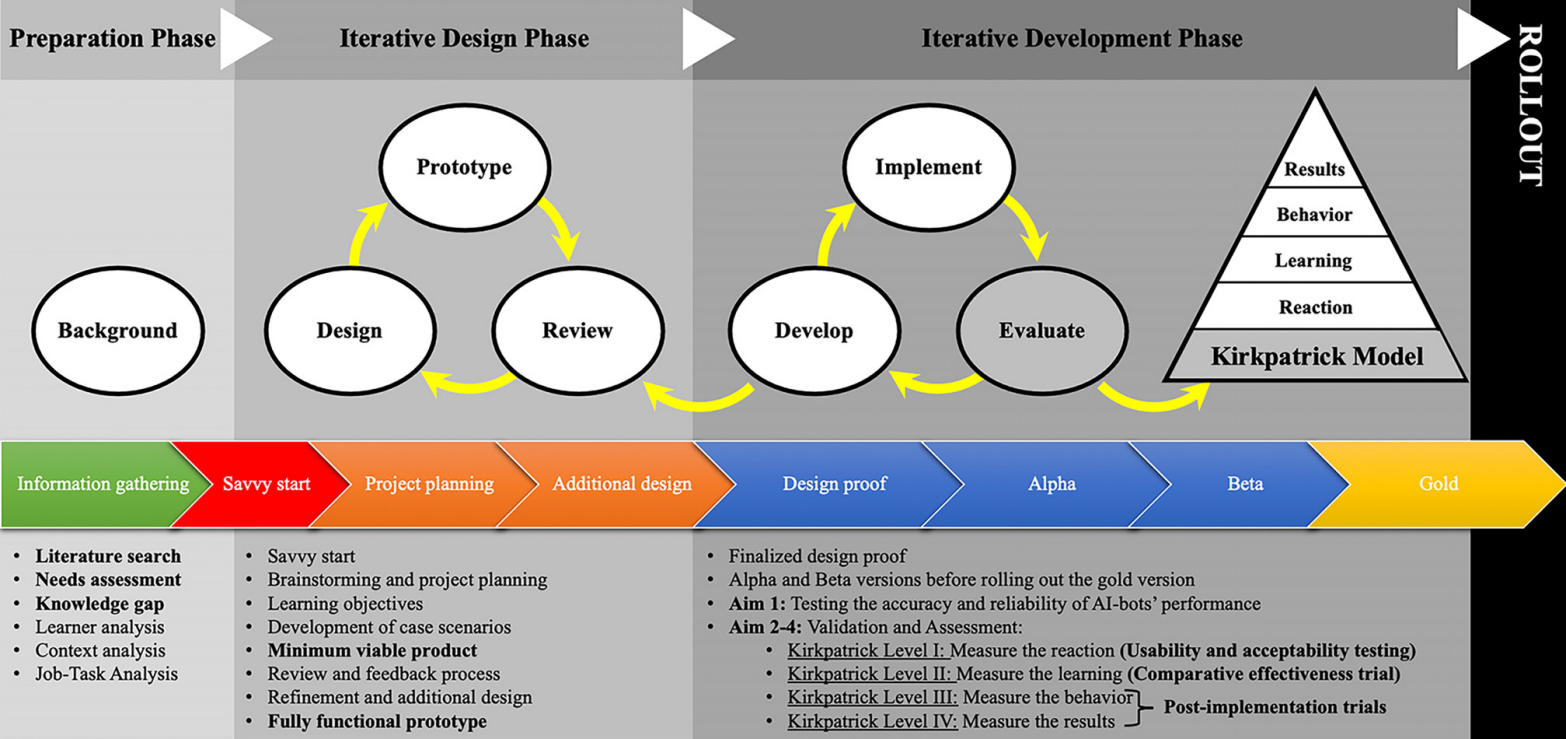
**SAM applied to the iterative development and evaluation of STOP.** This figure illustrates the iterative instructional design workflow that underpins the development of STOP, emphasizing rapid prototyping and continuous refinement through iterative cycles of design, review, and development. It depicts the progression from initial background and needs assessment and early “savvy start” activities to staged releases and rollout. Evaluation is aligned with the Kirkpatrick framework to facilitate systematic assessment throughout the implementation phases. Abbreviations: SAM: Successive Approximation Model; STOP: Standard Training and Operating Procedure.

The development of the STOP algorithm adhered to the principles of the Successive Approximation Model (SAM), a highly iterative approach designed for rapid prototyping and continuous refinement [10]. This model enabled us to create, test, and refine the algorithm in real-time, ensuring that each version aligned with real-world needs and evidence-based practices (Figure 2).

To establish a foundation, we conducted a comprehensive review of current guidelines, best practices, and expert recommendations regarding the management of mechanically ventilated patients. Following this, we initiated the “savvy start” phase of SAM. During this phase, a diverse, multidisciplinary team—including experts in critical care, respiratory therapy, pulmonary medicine, and clinical education—collaborated to develop a minimum viable prototype of the STOP algorithm. This prototype was designed to address the most common and clinically relevant scenarios in mechanical ventilation, providing bedside clinicians with a structured, easy-to-follow decision aid.

Throughout the iterative design and development phases of SAM, the prototype underwent seven rounds of review by a panel of subject matter experts. This panel, comprising critical care physicians, respiratory therapists, and nurse practitioners, provided feedback from their unique perspectives on frontline care. In each round, the experts evaluated the prototype against criteria such as:

**Clarity**: Is each step clear and easy to follow under high-pressure ICU conditions?

**Feasibility**: Can bedside providers realistically implement each step in diverse clinical settings?

**Clinical Relevance**: Does each component align with evidence-based practices and address common ICU challenges?

Feedback from each iteration led to modifications, ensuring that the algorithm was user-friendly, clinically relevant, and adaptable to various healthcare settings. For instance, steps were rephrased for clarity, redundancies were eliminated, and specific metrics (such as safe limits for ventilator settings) were incorporated to provide clear guidance on decision points.

By the conclusion of the iterative design and development phases, the STOP algorithm had evolved into a streamlined, eight-step tool tailored to address both routine and high-risk scenarios in mechanical ventilation management. Each step is designed to encompass essential actions, from assessing abnormal vital signs and ventilator alarms to evaluating patient readiness for spontaneous awakening and breathing trials, with the aim of standardizing care and reducing variability in practice. The collaborative and iterative nature of this process ensured that the STOP algorithm was grounded in both best practices and frontline feasibility, laying a strong foundation for further refinement and testing.

Following the development of the initial STOP algorithm prototype, we conducted a Modified Delphi process to enhance its clinical relevance and usability across various ICU environments. Utilizing the Checklist for Early Recognition and Treatment of Acute Illness and Injury (CERTAIN) Network [11], we engaged a global sample of multidisciplinary clinicians from 19 countries across 4 continents, who contributed their expertise in ventilator management through multiple rounds of feedback and discussion. In accordance with the Modified Delphi method, participants reviewed and rated each step of the algorithm to ensure practical feasibility and alignment with best practices in ventilator management. Each round included targeted questions to assess the effectiveness, clarity, and applicability of specific algorithm steps. Experts rated the feasibility and clinical relevance of each component, with a consensus defined as achieving at least 70% agreement among participants [12, 13].

Elements that did not initially reach consensus were revised based on participant feedback or removed from the algorithm in subsequent rounds. Following these rounds, we conducted a webinar with all stakeholders to review open-ended comments and feedback gathered during the Delphi process. In addition to making final revisions, we discussed optimal implementation strategies and future directions for the STOP algorithm, ensuring it would be both evidence-based and adaptable to diverse clinical settings. In accordance with Delphi methodology guidelines [13, 14], formal sample-size estimation was not applied, as Delphi techniques emphasize expert consensus rather than inferential statistics.

## Results

Thirty-three clinicians from diverse backgrounds participated in the iterative development and internal refinement of the STOP algorithm using SAM (Table 1). Over seven SAM review cycles, the multidisciplinary expert panel evaluated successive prototypes of the algorithm, refining each iteration based on frontline feasibility, clarity, workflow integration, and alignment with evidence-based practices in mechanical ventilation.

**Table 1:** Demographic characteristics of clinicians participating in iterative design/development phases (SAM) and external consensus phase (Modified-Delphi)

	**Design and development phase (SAM) (*n* ═ 33)**	**External consensus phase (Modified-Delphi) (*n* ═ 50)**
**Demographic characteristics**		
**Gender**		
Female	10 (30%)	18 (36%)
Male	23 (70%)	32 (64%)
**Profession**		
Physician	29 (88%)	43 (86%)
Nurse practitioner	1 (3%)	2 (4%)
Respiratory therapist	3 (9%)	5 (10%)
**Specialty distribution**		
Anesthesiology	7 (24%)	12 (24%)
Internal medicine	2 (7%)	19 (38%)
Pulmonology	16 (48%)	4 (8%)
Cardiology	0	2 (4%)
Surgery	0	3 (6%)
Pediatrics	0	2 (4%)
Emergency medicine	2 (7%)	0
Infectious disease	1 (3%)	0
Other	5 (17%)	8 (16%)
**Years of experience**		
<1	0	2 (4%)
1-5	7 (21%)	17 (34%)
6-10	6 (18%)	10 (20%)
11-15	6 (18%)	9 (18%)
Over 15	14 (43%)	12 (24%)

Abbreviation: SAM: Successive Approximation Model.

This iterative prototyping process culminated in a final 8-step procedure, guiding bedside providers through a structured algorithm and decision aid (Figures S1-S4):

- Are the vital signs abnormal and/or is the ventilator alarming?
- Is there adequate ventilation?
- Is the peak pressure high?
- Is the plateau pressure high?
- Are the lungs protected from ventilator-induced lung injury (VILI)?
- Is there a concern for oxygen toxicity?
- Is there patient-ventilator asynchrony?
- Is the patient ready for spontaneous awakening and breathing trials?

Throughout the SAM-based development phase, expert feedback informed targeted refinements, including the reorganization of step sequencing, clarification of decision prompts, standardization of terminology and thresholds, and the removal of redundancies. This process emphasized usability under real-world ICU conditions and facilitated the development of a coherent, clinically intuitive workflow.

Upon completion of the iterative development phase, the finalized STOP algorithm underwent external expert review using a Modified-Delphi consensus process involving 50 clinicians, which demonstrated high overall agreement across all eight steps (82%–96%), thereby supporting the face validity and clinical relevance of the final tool. Detailed algorithm flowcharts, operational thresholds, and supporting evidence are provided in the E-Supplement.

## Discussion

We present an iterative development of a structured, eight-step approach to support bedside decision-making for mechanically ventilated patients. By prioritizing relevant over irrelevant information, the STOP algorithm is designed to minimize errors and delays, reduce practice variability, and standardize care. A diverse group of clinician experts achieved a high degree of consensus across all components of the algorithm.

Previous efforts to develop decision aids and protocols for mechanical ventilation management have significantly contributed to the standardization of care and improvement of patient outcomes [2, 15]. A systematic review and meta-analysis by Parhar et al., which included more than 5,900 mechanically ventilated patients across 14 studies, demonstrated that standardized management of hypoxemic respiratory failure and acute respiratory distress syndrome (ARDS) was associated with a significant reduction in mortality and an increase in ventilator-free days compared to usual care [16]. Comprehensive ICU care frameworks, such as the PADIS (Pain, Agitation/Sedation, Delirium, Immobility, Sleep Disruption) guidelines [17] and the ABCDEF (Assess, Prevent, and Manage Pain; Both Spontaneous Awakening Trials (SAT) and Spontaneous Breathing Trials (SBT); Choice of Analgesia and Sedation; Delirium: Assess, Prevent, and Manage; Early Mobility and Exercise; Family Engagement and Empowerment) bundle [18], integrate mechanical ventilation within broader strategies to enhance overall patient outcomes. However, while these frameworks provide essential guidance for holistic ICU management, challenges remain in translating their recommendations into bedside decision-making tools that guide clinicians through the full spectrum of ventilatory management in real time. The STOP algorithm addresses this gap by integrating evidence-based [17] ventilatory strategies into a structured, stepwise approach, providing a streamlined and actionable tool to optimize mechanical ventilation and improve patient outcomes.

Protocols emphasizing spontaneous awakening and breathing trials have effectively reduced the duration of mechanical ventilation and ICU stays [19–23]. The study conducted by Pun et al. illustrated that full compliance with the ABCDEF bundle correlated with a significantly lower likelihood of requiring mechanical ventilation the next day (AOR, 0.28; 95% CI, 0.22–0.36). This underscores the essential role of sedation management, delirium prevention, and early mobility in facilitating timely liberation from mechanical ventilation [24]. The STOP algorithm builds on these principles by incorporating a structured decision tree for weaning. Unlike traditional weaning protocols, STOP treats weaning as an active component of a comprehensive algorithm, ensuring that clinicians systematically reassess readiness each time they ‘STOP’ at the bedside. This approach allows for continuous adaptation of ventilatory strategies to meet real-time patient needs.

Tools designed to guide tidal volume settings based on predicted body weight have streamlined the implementation of lung-protective ventilation strategies [25, 26]. The ARDS Network guidelines advocate for maintaining low tidal volumes (4–8 mL/kg of predicted body weight) while ensuring plateau pressure (Pplat) remains below 30 cm H2O to minimize VILI [27]. Positive End-Expiratory Pressure (PEEP) is also crucial in lung-protective ventilation, as it prevents alveolar collapse and reduces cyclic opening and closing injury. Higher PEEP strategies promote alveolar recruitment and diminish atelectrauma, particularly in patients with moderate to severe ARDS [28]. However, achieving a balance between recruitment and overdistension is vital, as excessive PEEP can result in hemodynamic compromise and overdistension of aerated lung regions. Recent studies have identified driving pressure (ΔP), defined as plateau pressure (Pplat) minus PEEP, as a key survival determinant in ARDS. Lower driving pressures, ideally below 15 cm H2O, are associated with improved outcomes and reduced mortality, rendering it an essential parameter in lung-protective ventilation [29, 30]. While these four pillars—low tidal volume, limited plateau pressure, optimized PEEP, and minimized driving pressure—form the foundation of lung-protective ventilation, mechanical ventilation can also induce lung injury due to excessive inspiratory effort, leading to patient self-inflicted lung injury (P-SILI) and diaphragmatic injury (myotrauma) [31]. In patients on spontaneous ventilation modes, such as Pressure Support Ventilation or Adaptive Support Ventilation, measuring the Pressure Measurement Index (PMI) and airway occlusion pressure (P0.1) is critical. Elevated values of PMI or P0.1 indicate increased inspiratory effort, which may exacerbate P-SILI and myotrauma [32, 33]. When excessive inspiratory effort is detected, adjusting ventilatory support or transitioning to controlled ventilation modes can mitigate these risks [34]. The STOP algorithm builds upon these lung-protective strategies by providing a comprehensive framework that incorporates evaluation of underlying etiology (Step 4) and facilitates dynamic adjustments of PEEP, driving pressure, and fraction of inspired oxygen (FiO_2_) to optimize lung protection (Steps 5–6). Additionally, STOP emphasizes assessing patient-ventilator interaction (Step 7), addressing asynchronies that can compromise both ventilation efficacy and patient comfort. This integration fosters a holistic approach to reducing VILI while enhancing synchronization between the patient and the ventilator, ultimately improving clinical outcomes.

Algorithms and automated systems designed to detect VILI or optimize ventilation settings have also shown promise [35, 36]. A study by Herasevich et al. demonstrated that an electronic algorithm for real-time monitoring and alerting of potentially injurious ventilator settings significantly reduced exposure to harmful ventilation settings from 40.6 ± 74.6 h to 26.9 ± 77.3 h (*p* ═ 0.004) [37]. This reduction illustrates the system’s effectiveness in influencing bedside practice and enhancing patient safety by minimizing VILI. While these systems provide precision and real-time capabilities, they often necessitate advanced technological infrastructure and substantial clinician training, which may limit their feasibility in resource-limited settings. In countries, such as the USA, Canada, and China, respiratory therapists are integral in continually assessing ventilator settings, serving as a human alternative to electronic surveillance. However, access to trained respiratory therapists is inconsistent across healthcare systems, particularly in low-resource environments. Conversely, the STOP algorithm presents a structured, intuitive approach that enhances decision-making at the bedside, making it particularly advantageous in ICUs with limited technological infrastructure or respiratory therapist availability.

While the STOP algorithm offers a standardized approach to mechanical ventilation management, several limitations warrant acknowledgment. First, the primary focus of this study is on developing and refining the STOP algorithm within the SAM framework in instructional design. The paper does not evaluate its clinical effectiveness or patient outcomes. Although the algorithm is based on expert insights and current evidence, further testing in real healthcare settings is necessary to determine its impact on ventilator practices, patient safety, and clinical outcomes. Second, we employed a Modified-Delphi consensus method to ensure the clinical relevance and face validity of the final algorithm. However, detailed psychometric analyses, such as inter-rater reliability measurements and round-by-round score changes, were not addressed in this article. These methodological aspects will be discussed in a separate manuscript focusing on the Delphi process. Consequently, the current results confirm content validity and usability but are not statistically definitive. Third, the findings may not be directly applicable to all ICU settings due to variations in resources, staffing, and care protocols. While a diverse group of clinicians contributed to the development, the feasibility of implementation may vary, especially in locations with limited respiratory therapy support or technology. Local adaptation of the algorithm and context-specific implementation strategies may be necessary. Additionally, the study did not formally assess human factors such as usability under high workload conditions or time constraints during development. Although STOP was designed as a user-friendly cognitive aid, empirical usability assessments are required to evaluate its performance in actual ICU workflows. Lastly, STOP is intended to assist clinicians in decision-making rather than replace human involvement. Its effectiveness hinges on clinician engagement, adherence, and appropriate application in clinical contexts. Variations in training, experience, and workflow integration can affect the consistency of algorithm use. To address these constraints, a prospective stepped-wedge cluster implementation and testing study (PIT-STOP) is currently underway (Figure 1). This study aims to evaluate real-world usability, adherence, and clinical impact in diverse ICU settings, incorporating continuous enhancements based on feedback from frontline clinicians.

## Conclusion

The STOP Project represents an innovative approach to improving mechanical ventilation management in critical care, bridging complexity with a practical solution necessary for effective bedside application. By providing a streamlined, evidence-based algorithm that emphasizes simplicity without sacrificing clinical relevance, STOP aims to standardize care, reduce variability, and ultimately enhance patient outcomes. The multi-phase development and implementation strategy ensures that STOP is adaptable to diverse healthcare environments and responsive to the needs of both experienced clinicians and trainees. Ultimately, the STOP project underscores a commitment to patient-centered, high-value care, with the goal of translating best practices into actionable bedside protocols that support ICU teams worldwide in delivering optimal respiratory support.

## Supplemental Data

Supplemental data are available at the following link: https://www.bjbms.org/ojs/index.php/bjbms/article/view/13288/4102.

## Data Availability

The data are available from the corresponding author upon reasonable request.
